# Effects of Contamination of Freshwater Habitat With Common Heavy Metals and Anions on the Prevalence of Human Adenoviruses and Enteroviruses

**DOI:** 10.3389/fpubh.2020.603217

**Published:** 2021-01-20

**Authors:** Michael Opere Wasonga, John Maingi, Ombori Omwoyo

**Affiliations:** ^1^Department of Biochemistry, Microbiology and Biotechnology, School of Pure and Applied Sciences, Kenyatta University, Nairobi, Kenya; ^2^Department of Plant Sciences, School of Pure and Applied Sciences, Kenyatta University, Nairobi, Kenya

**Keywords:** environmental monitoring, adenoviruses, enteroviruses, heavy metals, anions, freshwater

## Abstract

The occurrence and survival of enteric viruses in open surface waters can be impacted by a host of factors including fecal emission levels, seasonal variations, virus stability and the physicochemical parameters. In this research, we aimed to document the association between contaminations of water samples with human enteric viruses (adenoviruses and enteroviruses) from a freshwater lake with variations in chemical contaminants. We collected 216 water samples from October 2010 to April 2012, from a 4 km stretch along Lake Victoria (LV) basin in Homa Bay town located in the western region of Kenya. The samples were analyzed for the existence of human adenoviruses (HAdV) and human enteroviruses (HEV), using the nested PCR (nPCR). We also assessed in the water samples the levels of twelve chemical contaminants consisting of six heavy metal elements and six anions. About 8.3 % of the samples were found to be contaminated with the enteric viruses. The concentrations of the 12 chemical contaminants were found to be largely within the WHO suggested limits. Most of the chemical contaminants were not related to the detection rates of the viruses from the statistical analysis. However, some positive and negative associations between the viral genome's detection and the chemical concentrations were established for only three metals (Fe, Pb, Cd) and the PO4^3−^ Radical. Cd had a weak positive significant relationship with HAdV (rho = 0.146, *p* = 0.032) while Pb and Fe had a weak positive significant relationship with HEV genome detection (rho = 0.156, *p* = 0.022) and (rho = 0.148 and *p* = 0.029) respectively. There was a modest negative relationship between phosphate ions and HEV (rho = −0.174, *p* = 0.010). The results of our study do not provide support for the hypothesis of an association between the presence of human enteric viruses and the levels of twelve chemical contaminants.

## Introduction

Water quality issues have escalated over time globally in response to increased population growth and the corresponding socio-economic activities (1). Kenya's freshwater ecosystems, just like in other low- and middle-income countries have been increasingly at risk of chemical contamination in recent years, due to rapid demographic changes, which coincided with the development of human settlements accompanied by the limited establishment of adequate sanitary infrastructure (2). Lake Victoria (LV) is one of the significant freshwater ecosystems in Kenya that serves part of the East African population. Changes in land use exacerbated by agricultural and industrial development have led to the contamination of LV by different sets of pollutants, including chemical contaminants. Chemically contaminated water supplies can directly impact the health of the contact populations as well as act as a reservoir for incidence and survival of potentially pathogenic microorganisms such as enteric viruses (3). Microbial communities are usually one of the biota commonly directly affected by various chemical pollutants including anions and heavy metals (4). Different chemical contaminants have been reported to affect bacterial, fungal, archaea, and protozoan communities in various aquatic ecosystems (5).

The impacts could be either beneficial or detrimental to the microbe depending on the concentration and the form of existence of the chemical contaminant at issue. Some of the contaminants play crucial roles in the micro-organisms' metabolic and physiological processes. They are not only micronutrients to the microbes but also useful for structural function, electrostatic interactions, molecular stability, regulation of osmotic pressure, and enzymatic activity as cofactors among other biochemical reactions (5, 6). Some studies have documented that concentrations of most heavy metals at low levels are essential for various metabolic activities and the maintenance of ionic concentrations in various microbes (5). On the contrary, exposure to higher concentrations can result in negative effects such as osmotic imbalance, and alterations in the microbe's structure. These negative interference may subsequently lead to a decrease in diversity, prevalence, biomass and distribution of the microbes (7). Consequently interference with the microbial community profile may negatively or positively affect the ecosystem balance. The positive outcome can be linked for example to improvements that could reduce an infectious pathogen's population while negative results may be linked for example to conditions that decrease the ecological activities associated with the optimum concentration of microbes in the environment such as organic matter decomposition, nitrification and denitrification (8). Exposure to elevated concentrations of some heavy metals and non-metals has also been reported to have adverse effects to the contact population. Various chemicals contaminats have been associated with diseases, disorders and complications of the major organs and organs such as lung, brain, skin, kidney, liver, pancreas, nervous, respiratory, and immune systems (9–11).

Several specific mechanisms of action have been reported on the action of the chemicals on the microbes. Heavy metals such as Mercury (Hg), Lead (Pb), Cadmium (Cd), Zinc (Zn), Nickel (Ni), Copper (Cu), and Arsenic (As) can desaturate the protein, inhibit cell division, enhance transcription and the enzymatic activity, and induce nucleic acid damage (11–13). Research on the impact of chemical environmental pollution and interaction with bacterial communities has been extensively studied (13). However, few studies have concentrated on elucidating the interaction of viruses with the concentration of heavy metals and non-metals in the water environment especially in the Sub Saharan Africa. Jurzik et al. (14) reviewed the correlation between the occurrence of human adenovirus, rotavirus and human polyomavirus and some anions in Germany. They found that the concentrations of the anions studied had not significantly affected the occurrences of any of the enteric viruses groups. A research elsewhere by Vecchia et al. (15) was corroborative to the findings, however they reported a weak positive correlation between human adenoviruses and the heavy metals; cadmium, iron, and chromium, as well as with nitrate radical. Similarly some of these metals have been found to influence viruses' survival on different fomites (16). Notably, Copper (Cu) can have negative effects on certain viruses, such as Noroviruses.

These effects could be attributed to different mechanisms of action of the chemicals on the virus's biochemical processes ranging from binding to viral proteins to the activation of the reverse transcription process. Some ions typically form an integral part of viral proteins where they play significant roles in the survival and pathogenesis of the virus. Some heavy metals such as zinc and copper ions bind to the viral proteins where they are involved in different activities such as genomic material maturation, activation and catalytic activity, reverse transcription, initial integration and defense of newly synthesized DNA materials (17). Non-metals such as chlorine have been reported to deactivate the cellular materials of the virus through oxidation (18). They can also inhibit translocation of protons, boost annealing of the nucleic acid, get involved in the incorporation of the viral DNA into specific sites, and in the formation of integral part of nucleic acid. This study highlights the significance of different chemical components in the occurrence of enteric viruses in an open freshwater water source. The data may be useful in informing early design and development of initiatives to counter environmental potentially pathogenic viral contamination (17).

Different types of enteric viruses have been recovered from various environmental water matrices including freshwater habitat (19). These viruses are of public health importance, because of their link to infections such as gastroenteritis in communities from poor sanitation settings (20). Adenoviruses and enteroviruses are among the most important groups of enteric viruses in public health due to their ubiquity and stability in aquatic environments (21). Adenoviruses are non-enveloped, double-stranded DNA viruses of the *Adenoviridae* family and the *Rowavirales* order, consisting of over 80 distinct human serotypes (22). Enteroviruses, on the other hand, are non-enveloped single-stranded RNA viruses and belong to the *Picornaviridae* family and order *Picornavirales* with more than 100 known human serotypes (23). Even though they have been isolated from various environmental water matrices, their relative abundance was substantially high in urban settings because of the pollution with sewage effluents and associated wastewater discharge (24).

Fecal contamination analysis has primarily been used to signal for the potential existence of these viruses in surface waters (25). Some studies have however reported that chemical contaminants such as phosphates, nitrates, and heavy metals could also be used as indicators and predictors of these viruses' presence and stability in environmental waters. Some of these compounds have metabolites that could be excreted with metabolic waste and end up in surface waters (26). Our goal was therefore to assess the potential for use of chemical pollution as potential predictors for the occurrence of potentially pathogenic enteric viruses in freshwater habitat with respect to human enteroviruses (HEV) and human adenoviruses (HAdV).

We previously investigated the correlation between the occurrences of these viruses with variations in physical quality water quality characteristics (27). In the present study, we further focus on the association between specific surface water chemical attributes and enteric virus contamination. The research was performed in the western region of Kenya from the open waters of LV in Homa Bay town (HB). The chemical contaminants considered comprised of six heavy metals namely Iron (Fe), Cadmium (Cd), Zinc (Zn), Lead (Pb), Copper (Cu) and Mercury (Hg) and six anions that is Chlorides (Cl^−^), Carbonates (${\text{CO}}_{3}^{2-}$), Sulfates (${\text{SO}}_{4}^{2-}$), Fluorides (F^−^), Nitrates (${\text{NO}}_{3}^{-}$) and Phosphates (${\text{PO}}_{4}^{3-}$). The average concentrations of the chemical pollutants assessed were also compared with the standards prescribed by the WHO (28) for environmental waters to determine the quality and suitability of the waters for human use.

Environmental waters chemical contamination pathways may include surface runoff, leaching and water seepage through tailing piles from mines and other anthropogenic sites as well as from natural mineralized deposit zones (29). Various studies elsewhere have documented the impact of these chemicals on freshwaters and their relationship to enteric viruses' contamination (14, 30, 31). Despite the study area being characterized by a multiple of anthropogenic activities that could be linked to sources of surface water pollution with different chemical and viral contaminants, no relevant research has been reported on the same. Achieng et al. (32) and Chebet et al. (33) reported on the concentration of some of these chemical contaminants from some water sources in Kenya. However, these studies were focused about chemical pollution only and did not attempt to link the results with enteric virus contamination of the source waters.

## Materials and Methods

### Study Area

**Figure 1:**
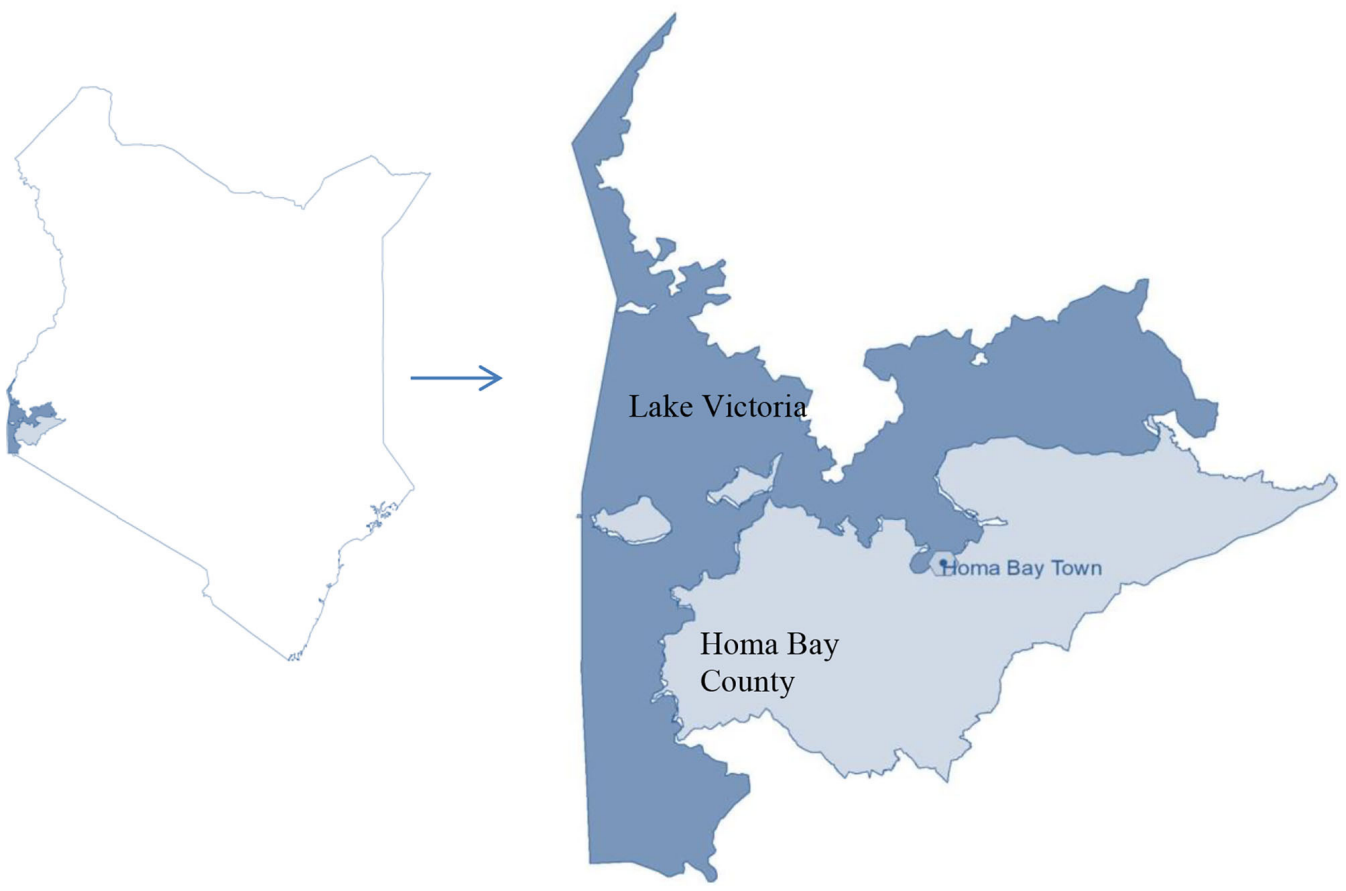
Map of Kenya showing Homa Bay County and the town.

**Figure 2:**
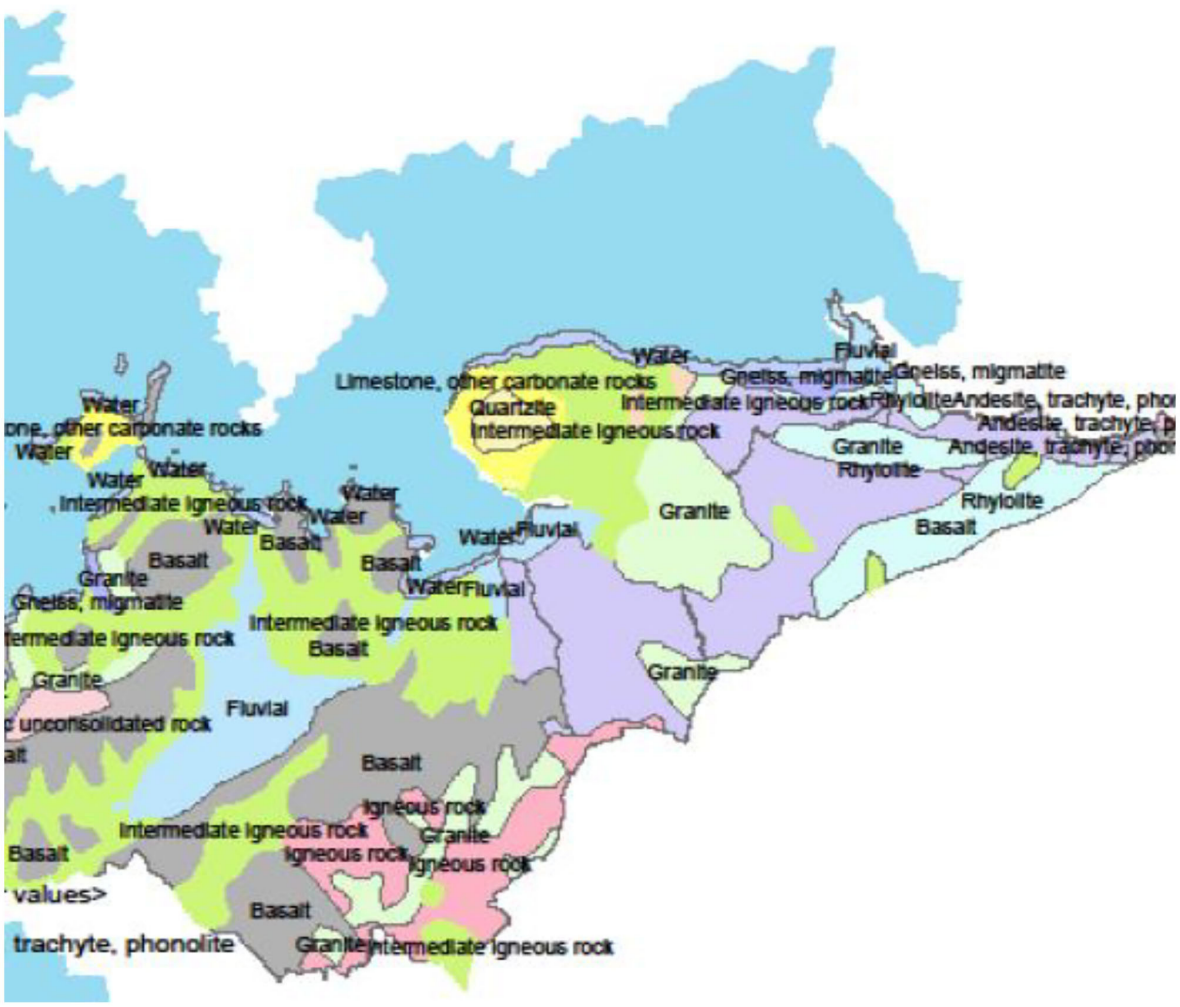
Map of Homa Bay County showing the lithography (36).

The study was undertaken in Homa Bay town situated in the western region of Kenya at decimal −0.5389228, 34.4137494 in Homa Bay County (Figure 1). The town has a size of ~29 km^2^ (34), with an estimated population of 45,000 (35). The town and its surroundings are characterized by many anthropogenic activities including horticulture, water transportation, car washing, and industrialization (36). In addition to the pollution likely to be caused by urbanization and industrialization in the town, the regional geological structure favors deposits of minerals that may contain the mineralized forms of the chemical contaminants, thus raising the chances of pollution of the lake waters through erosion and underground reservoirs. Artisanal mining of some of the mineral deposits has been on going along the lakeshore for more than a decade thus increasing the chances of contamination. For example, iron ore mining around the lakeshore at Homa Hills region (36) might contribute to Fe contamination. Other mineral deposits in the region include limestone at Homa Hills, Gold at Rangwe and Kendu Bay, soda ash at Homa hills, niobium and phosphate at Homa Hills and Gwasi (36, 37). Figure 2 provides the regional map showing the locations of the mineral deposits and the general lithology around the lake. In addition to gold mining, deposits of zinc and copper have been discovered in the neighboring Migori County, which is also located along LV's shores. It has been reported from the Gold mining activity at Macalder in Migori County that Mercury was used for amalgamation. This could contribut to contamination of the lake waters with Hg through the receiving waters (38). The quality standard of the town's surface waters and the surrounding area has been compromised and aggravated by a range of factors, including inadequate treatment system, poor urban drainage system as well as the rolling topography of the area. In essence, the gentle rolling topography significantly heightens urban runoff to the lake shore (34).

### Samples Collection

**Figure 3:**
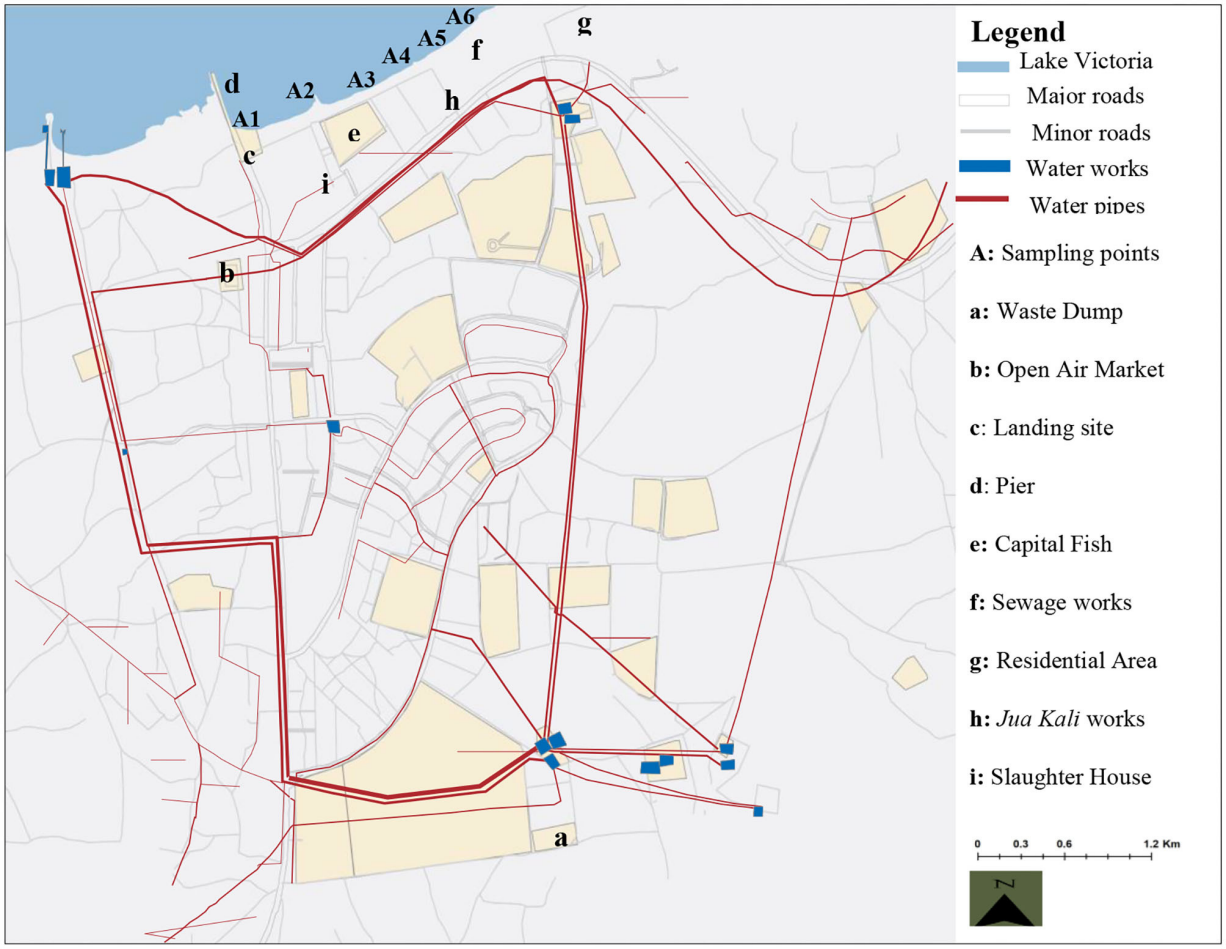
Map of Homa Bay town showing the sampling sites, water infrastructure and the orientation to the possible sources of contaminants (34).

The actual sampling points in the town were distributed within the Central Business District covering a stretch measuring ~9 km^2^. The sampling strip was selected from an area oriented to the identified potential onsite sources of contamination (Table 1). The surrounding region is characterized by high level of different forms anthropogenic activities including general agricultural, fisheries, water transport, commerce, recreation and wastewater treatment activities (Figure 3). A total of six sampling points designated as A1–A6 were identified at different sections along the strip (Figure 3). Water samples were collected into sterilized 10-L polyethylene containers and transported on ice to the enteric viruses' research laboratory at the Institute of primate research in Nairobi for viral analysis. Similarly, an additional 1 L of the samples was collected from the same spots for chemical analysis for a period of 6 months from October 2011 to April 2012. The total number of samples collected for both analyses were 216 (*n* = 6 per site in a sampling trip).

**Table 1:** Description of the potential sources of chemical contamination and their physical/global positioning system (GPS) locations.

**Source**	**Physical/GPS location**	**Potential contaminant (analyte)**
Soda ash deposit/mines	Homa Hills	${\text{CO}}_{3}^{2-}$
Limestone/mines	Homa Hills	${\text{CO}}_{3}^{2-}$
Iron ore deposit/mines	Homa Hills	Fe
Gold deposits/mines	Kendu Bay, Rangwe, Migori County	Cd, Hg, Zn, Pb, Cu ${\text{SO}}_{4}^{2-}$
Phosphate deposits	Homa Hills	${\text{PO}}_{4}^{3-}$
Zinc deposits	Migori County	Zn
Copper deposits	Migori County	Cu
Water works	HB/−0.52214, 34.46304	Cl^−^
Sewage treatment plant	HB/−0.52149, 34.46193	${\text{NO}}_{3}^{-}$ and Cl^−^
Horticulture	Along the lake shore	${\text{PO}}_{4}^{3}$, Cl^−^and ${\text{NO}}_{3}^{-}$
Water and Road transport	Landing zone/HB-Kisumu Highway	Pb
Car washing	HB/−0.52134, 34.45994	Pb
Jua Kali Industry	HB/−0.52236, 34.46043	Pb, Cd, Zn, ${\text{SO}}_{4}^{2-}$
Capital Fish Industry	HB/−0.52380, 34.45779	${\text{NO}}_{3}^{-}$
Slaughter House	HB/−0.52455, 34.45738	${\text{NO}}_{3}^{-}$
Water reticulation network	Within HB	Zn, Cl^−^, Cd
Waste Dump site	Homa Bay town	${\text{NO}}_{3}^{-}$, ${\text{SO}}_{4}^{2-}$, ${\text{PO}}_{4}^{3-}$, Cd, and Pb
Animal feeds industry	HB/−0.52222, 4.45942	${\text{NO}}_{3}^{-}$

### Analysis of the Samples for the Chemical Contaminants

We analyzed the water samples for contamination with the 12 different chemical contaminants comprising of six heavy metals (Iron, Cadmium, Copper, Lead, Zinc, and Mercury) and six anions (chlorides, fluorides, sulfates, nitrates, phosphates, and carbonates) following the methods adopted from the standard methods for the examination of water and waste water (39). Briefly, the heavy metals Fe, Cd, Pb, Cu, Zn, and Hg were analyzed using the Atomic Absorption Spectrometer (AAS) Buck Scientific (210 VPG) (air—acetylene flame). Similarly, spectrophotometry techniques were used to analyze the anions. Fluorides were analyzed using SPADNS methods, sulfates using the turbidimetric methods, phosphates using the ascorbic acid methods, nitrates using the colorimetric techniques, chlorides using argentometric methods while the potentiometric methods were used for bicarbonates analysis. Spectrophotometric wavelength readings in all cases were taken against the standard solution**s** and recorded. The Standard solutions were prepared using specific analar grade reagents for each of the chemical elements to be analyzed. Each specific analar was dissolved in distilled water from which calibration curves were developed before analyzing the water samples.

### Analysis of the Samples for Viral Contaminants

Viral genomes recovery were carried out using the glass wool adsorption-elution technique from the protocols previously used by Vilaginès et al. (40), Wolfaardt et al. (41) and Lambertini et al. (42). The whole of the water sample for viral analysis (10 L) was passed through a column of oiled sodocalcic glass wool filters (Bourre 725 QN, Ouest Isol, Alizay, France) by applying negative pressure using a Vacuum pump. Glycine beef extract buffer (GBEB) pH 9.5 (Merck GmbH, Darmstadt, Germany) was used to elute the virus particles that got adsorbed to the glass wool column into sterile glass tubes. The eluates were further subjected to secondary concentration by precipitating the eluate using Polyethylene glycol (PEG)/Sodium Chloride (NaCl) (Merck GmbH, Darmstadt, Germany). Secondary concentrates were subjected to an overnight incubation at 4°C followed by centrifugation for 45 min at 4,200 rpm (Eppendorf 5,402 microcentrifuge; Eppendorf Gerätebau, Netheler and Hinz GmbH, Hamburg Germany). Phosphate-buffered saline solution [Sigma-Aldrich (St. Louis, MO)] was used to resuspend the resultant pellets for another round of centrifugation from which the resultant supernatant was used for nucleic acid extraction. The concentrates were aliquoted in sterile glass tubes and temporarily stored at −70°C until use. Quality control during the processing was enhanced through careful handling of the laboratory procedures and adopting different laboratories during different rounds of sample processing. A new set of laboratory apparatus was used each time and within the different laboratories to minimize the probability of cross-contamination.

From the secondary concentrates, 1 mL was used for DNA extraction using an automated commercial MagNA Pure total DNA extraction kit (Large Volume) (Roche Diagnostics). Similarly, 2 mL of the concentrates were used for RNA extraction using the RNeasy mini kit (QIAGEN) following the manufacturer's protocols. Uracil DNA glycosylase was used to treat the nucleic acid extracts to reduce the probability of amplifying contaminant DNA. PCR process was adopted from the methods originally described by Puig et al. (43) using nested primer pairs as previously used by Allard et al. (44), Allard et al. (45) and Santos et al. (46). cDNA was synthesized by Reverse Transcription (RT) from the RNA extracts using a Reverse transcriptase-SuperScript II (Invitrogen, Carlsbad, CA), 20 U of RNasin (Promega). Positive and negative controls were introduced alongside the samples during the PCR process. Positive control for adenoviral genome was derived from HAdV-C2 human adenovirus strain (ATCC VR-1079 AS/Rab) while that of enterovirus was derived from poliovirus type 1. Nuclease free water was used as a negative control. The remaining nucleic acid extracts were stored at −20 and −80°C for DNA and RNA extracts, respectively, until use. Following the PCR process, agarose gel electrophoresis (2%) stained using ethidium bromide was used to visualize the products. Samples were analyzed for PCR inhibition by spiking some of the aliquots that had returned positive and negative results with each of the specific virus positive control for another round of PCR process.

### Data Analysis

Descriptive statistics were used to analyze pollution of the waters from the six sites with the chemical contaminants. Spearman's correlation coefficient was used to analyze the association between the 12 chemical pollutants and the presence of the enteric viruses. Logistic regression analysis was also performed to determine the probability that the chemical contaminants would affect the frequency of the viruses' detection. All statistics were conducted using the program SPSS 20.0.

## Results

### Enteric Viruses' Contamination

The two classes of enteric viruses examined were detected from all sampling sites along the study stretch, with overall detection rate being 8.33% (18/216) (Table 2). Of the positive samples, HAdV was at 5.09% (11 of 216 samples) while detection of HEV was 3.24 % (7 of 216 samples) A comparatively higher percentage of viral genome detection was reported from the sampling points to the east of the stretch (A5 and A6), with a total contamination percentage of 6.02% (13 of 216 samples) for the two sets of viruses. At A4 where low population density characterized the surrounding area with reduced human activity, only HEV was reported at 0.046% (1/216) while HAdV was not detected at all. HAdV detection was at 0.93% (2/216) from the other middle region sampling point (A3), while HEV detection was not detected. Like the points in the middle stretch, points located in the lower stretch (A1 and A2) also showed mixed results for both sets of viruses. Adenovirus contamination was detected only at A1 at a lower rate of 0.46% (1/216) while enteroviruses were detected only from site A2 at an equally lower rate of 0.46% (1/216).

**Table 2:** Detection rates of the viral genomes by site.

	***n***	**A1**	**A2**	**A3**	**A4**	**A5**	**A6**	**Positive samples (%)**	**Negative samples (%)**
HAdV	216	1 (0.46)	0 (0.00)	2 (0.93)	0 (0.00)	5 (2.31)	3 (1.39)	11 (5.09)	198 (94.91)
HEV	216	0 (0.00)	1 (0.46)	0 (0.00)	1 (0.46)	3 (1.39)	2 (0.93)	7 (3.24)	209 (96.76)
Total	216	1 (0.46)	1 (0.46)	2 (0.93)	1 (0.46)	8 (3.70)	5 (2.31)	18 (8.33)	407 (91.67)

### Chemical Contaminants' Concentration

In Table 3, the description of concentration by site findings and the overall descriptive statistics of the 12 chemical contaminants are shown. Whereas, contaminants concentration levels differed among the sampling points, all the recorded concentrations were lower than levels recommended by the WHO. For example, in samples from site A5 the concentration of Pb was higher at an average of 0.0039 mg/L. This was however far below the WHO's standard amounts at 0.01 mg/L. Similarly the average site-wide concentration of Hg was 0.000023 mg/L, which is significantly below the WHO permissible limit of 0.006 mg/L. The highest mean concentrations recorded for Hg were from sites A3 and A4 at 0.00003 mg/L. Concentration of some chemicals from some samples were however noted to be above the WHO limits as depicted by the recorded maximum values. Notably, the recorded maximum concentration for Iron and phosphate were above the WHO values of 0.3 mg/L and 0.5 mg/L, respectively. The lowest detection for Fe was however reported to be 0.1181 mg/L at point A2 while lowest phosphate concentration was 0.1381 mg/L at site A6. Although the average values of the contaminants per site were computed, it is noteworthy that some samples had reported values below the detection limits of the contaminants. For instance, some samples were below the phosphate detection limit which was 0.1 mg/L (Table 3).

**Table 3:** Concentration of the chemical contaminants by sampling points.

**Parameter (mg/L)**	**A1**	**A2**	**A3**	**A4**	**A5**	**A6**	**Combined descriptive statistics**				**WHO Limits**
							**Mean**	**SD**	**Min**	**Max**	
Fe	0.1278	0.1181	0.1361	0.1244	0.1231	0.1286	0.1263	0.07802	0.03	0.80	0.3
Cd	0.0002	0.0002	0.0001	0.0001	0.0007	0.0006	0.000308	0.0002514	0.0001	0.0009	0.003
Zn	0.0082	0.0084	0.0074	0.0072	0.0087	0.0079	0.007968	0.0014694	0.0050	0.0150	-
Cu	0.0817	0.0892	0.0750	0.0836	0.1019	0.0922	0.0873	0.05031	0.03	0.60	2
Hg	0.00002	0.00002	0.00003	0.00003	0.00002	0.00002	0.000023	0.0000072	0.0000	0.0000	0.006
Pb	0.0034	0.0029	0.0030	0.0027	0.0039	0.0036	0.003257	0.0008633	0.0015	0.0066	0.01
Cl^−^	14.7222	15.8611	12.3056	14.1389	20.8611	17.8056	14.972	4.7012	5.0	26.0	250
F^−^	0.0211	0.0339	0.0297	0.0264	0.0203	0.0242	0.0259	0.01519	0.01	0.09	1.5
${\text{CO}}_{3}^{2-}$	33.3333	43.6944	57.4167	69.5833	65.2778	60.8056	55.019	28.4252	25.0	230.0	-
${\text{NO}}_{3}^{-}$	9.0000	11.1111	10.3611	12.7778	15.2222	13.7222	12.032	4.0063	5.0	28.0	45
${\text{PO}}_{4}^{3-}$	0.1578	0.1439	0.1653	0.1811	0.1400	0.1381	0.1544	0.09020	0.05	0.90	0.5
${\text{SO}}_{4}^{2-}$	21.1111	20.0556	11.7778	13.0278	11.1111	19.8681	16.157	7.2416	6.0	48.0	400

### Relationship Between the Chemical Contaminants' and the Viral Genome Detection

The relationship between concentrations of the 12 chemical contaminants in the water sample was examined to tests if they correlated with the presence of the two viruses (HAdV and HEV) using the Spearman's Correlation Coefficient. The results showed that all the heavy metals; Fe, Pb, Zn, Cu and Hg had no major association with an adenoviral gnome except for Cd. There was, for example, a small, negative, insignificant correlation between Fe and HAdV (rho = −0.120, *p* = 0.077). Cd on the other hand, had a weak significant positive relationship with HAdV (rho = 0.146, *p* < 0.05). Only Pb and Fe showed a weak significant relationship with the detection of HEV genomes (rho = 0.156, *p* < 0.05) and (rho = 0.148 and *p* < 0.05) respectively. All the other 5 heavy metals did not record any significant relationship with HEV (Table 4). Similarly, all of the examined anions (Cl^−^, ${\text{CO}}_{3}^{2-}$, ${\text{SO}}_{4}^{2-}$, F^−^, ${\text{NO}}_{3}^{-}$, and ${\text{PO}}_{4}^{3-}$) showed no significant correlation to the occurrence of HAdV (*P* > 0.05). Equally, there was no substantial anions correlation to the HEV except for phosphate ion (${\text{PO}}_{4}^{3-}$) in which a mild negative association existed (rho = −0.174, *p* < 0.05) (Table 4).

**Table 4:** Spearman correlation of heavy metal and anion parameters and the viruses.

**S/No**	**Chemical contaminants**	***n***	**HAdV**	**HEV**
1	Fe	216	−0.120	0.148^*^
			0.077	0.029
2	Pb	216	0.079	0.156^*^
			0.246	0.022
3	Cd	216	0.146^*^	0.090
			0.032	0.186
4	Zn	216	−0.027	0.085
			0.697	0.212
5	Cu	216	0.029	0.093
			0.669	0.174
6	Hg	216	−0.106	0.028
			0.119	0.687
7	Cl^−^	216	−0.059	0.132
			0.385	0.053
8	F^−^	216	0.016	−0.109
			0.814	0.110
9	${\text{CO}}_{3}^{2-}$	216	0.045	0.025
			0.509	0.713
10	${\text{NO}}_{3}^{-}$	216	0.007	−0.025
			0.915	0.716
11	${\text{PO}}_{4}^{3-}$	216	0.012	−0.174^*^
			0.856	0.010
12	${\text{SO}}_{4}^{2-}$	216	−0.081	−0.005
			0.233	0.941

*Cell contents: Correlation Coefficient (rho); Probability value (P)*;

* *Correlated parameters with some level of statistical significance (p < 0.05)*.

Binary logistic regression analysis was carried out to determine the probability of using the 12 chemical pollutants as predictors for the existence or absence of the waters' viral contamination. All the model's fitting regression equations are tabulated in Table 5. From the model's fitting regression equation for the HAdV and the heavy metals, the log of the occurrence of HAdV was positively related to the concentration of Cd (*p* < 0.05) according to the model (Table 6). In other words, the higher the concentration of Cd, the greater the chance of HAdV occurring. Similarly from the model fitting regression equation for HEV and the heavy metals, the frequency of HEV detection was related positively to concentration of Pb (*p* < 0.05) (Table 7). This indicates the possibility that higher concentrations of Pb could increase HEV activity. Hosmer and Lemeshow test showed no significance suggesting that the model was a good fit model (Table 7). For HAdV and non-metals, the predicted logits indicate that there was no statistically relevant contribution to the model by any of the non-metals (Table 8). On the other hand, the predicted logit for HEV and non-metals indicated that the log of the probability of water contamination with HEV was negative with phosphate (${\text{PO}}_{4}^{3-}$) concentration (*p* < 0.05) but statistically insignificant for the other 5 predictors (*p* > 0.05) (Table 9). In other words, the higher the ${\text{PO}}_{4}^{3-}$ concentration, the less probable the HEV could contaminate the waters.

**Table 5:** Fitting regression prediction equations between the viruses and the predictor variables.

**Variables**	**Equations**
HAdV/Metals	HAdV = *logit*(*Y* = 1) = *b*_0_ + *b*_1_*Fe* + *b*2*Pb* + *b*3*Cd* + *b*4*Zn* + *b*5*Cu* + *b*6*Hg*
	*logit*(*Y* = 1) = − 0.949 − 12.473*Fe* + 399.5*Pb* + 2510.5*Cd* − 294*Zn* + 7.1*Cu* − 56777*b*6*Hg*
HAdV/Anions	HAdV = *logit*(*Y* = 1) = *b*_0_ + *b*_1_*Chloride* + *b*2*Fluoride* + *b*3*arbonate* + *b*4*Nitrate* + *b*5*Phosphate* + *b*6*Sulfate*
	*logit*(*Y* = 1) = − 0.753 − 0.066*Chloride* − 13.225*Fluoride* + 0.007*Carbonate* − 0.011*Nitrate* − 1.634*Phospahte* − 0.062*Sulfate*
HEV/Metals	*HEV* = *logit*(*Y* = 1) = *b*_0_ + *b*_1_*Fe* + *b*2*Pb* + *b*3*Cd* + *b*4*Zn* + *b*5*Cu* + *b*6*Hg*
	*logit*(*Y* = 1) = − 14.546 + 21.849*Fe* + 1365.281*Pb* + 392.394*Cd* + 91.499*Zn* + 15.202*Cu* + 47732.08*Hg*
HEV/Anions	HEV = *logit*(*Y* = 1) = *b*_0_ + *b*_1_*Chloride* + *b*2*Fluoride* + *b*3*Carbonate* + *b*4*Nitrate* + *b*5*Phosphate* + *b*6*Sulfate*
	*logit*(*Y* = 1, 0) = 1.047 + 0.118*Chloide* − 89.206*Fluoride* + 0.011*Carbonate* − 0.096*Nitrate* − 36.198*Phospahte* + 0.043*Sulfate*

**Table 6:** Logistic regression analysis of the 6 heavy metals and HAdV.

**Predictor**	***B***	***S.E*.**	**Wald's *χ2***	***df***	***Sig*.**	**Exp (*B*)**
Fe	−12.473	7.736	2.599	1	0.107	0.000
Pb	399.520	533.455	0.561	1	0.454	3.232E3
Cd	2510.514	1225.125	4.199	1	0.040	.
Zn	−294.130	298.688	0.970	1	0.325	0.000
Cu	7.095	8.855	0.642	1	0.423	1205.413
Hg	−56777.929	39676.018	2.048	1	0.152	0.000
Constant	−0.949	2.685	0.125	1	0.724	0.387

*Cox and Snell R2 = 0.054. Nagelkerke R2 = 0.164*.

**Table 7:** Logistic regression analysis of the 6 heavy metals and HEV.

**Predictor**	**B**	**S.E**.	**Wald**	**Df**	**Sig**.	**Exp (B)**
Fe	21.849	11.333	3.717	1	0.054	3081513717.143
Pb	1365.281	643.649	4.499	1	0.034	.
Cd	392.394	1591.470	0.061	1	0.805	2.599E + 170
Zn	91.499	250.891	0.133	1	0.715	5.462E + 39
Cu	15.202	11.990	1.608	1	0.205	4002075.783
Hg	47732.080	59260.752	0.649	1	0.421	.
Constant	−14.546	4.751	9.373	1	0.002	0.000
**Test**			***χ2***	***Df***	***p***	
**Goodness of fit test**						
Hosmer and Lemeshow			7.538	8	0.480	

**Table 8:** Logistic regression analysis of the six non-metals and HAdV.

**Predictor**	***B***	**S.E**.	**Wald's χ2**	***df***	**Sig**.	**Exp (*B*)**	**95% C.I. for EXP (*****B*****)**	
							**Lower**	**Upper**
Cl^−^	−0.066	0.074	0.788	1	0.375	0.936	0.810	1.083
F^−^	−13.225	23.430	0.319	1	0.572	0.000	0.000	1.6*10^14^
${\text{CO}}_{3}^{2-}$	0.007	0.011	0.452	1	0.501	1.007	0.986	1.028
${\text{NO}}_{3}^{-}$	−0.011	0.082	0.017	1	0.895	0.989	0.842	1.162
${\text{PO}}_{4}^{3-}$	−1.634	4.766	0.117	1	0.732	0.195	0.000	2.0*10^3^
${\text{SO}}_{4}^{2-}$	−0.062	0.054	1.350	1	0.245	0.939	0.846	1.044
Constant	−0.753	1.971	0.146	1	0.702	0.471		

*Cox and Snell R2 = 1.4%. Nagelkerke R2 = 4.3%*.

**Table 9:** Logistic regression analysis of the six non-metals and HEV.

**Predictor**	***B***	**S.E**.	**Wald's *χ2***	***df***	**Sig**.	**Exp (*B*)**	**95% C.I. for EXP (*****B*****)**	
							**Lower**	**Upper**
Cl^−^	−0.118	0.088	1.827	1	0.176	1.126	0.948	1.336
F^−^	−89.206	54.034	2.726	1	0.099	0.000	0.000	1.8*10^7^
${\text{CO}}_{3}^{2-}$	0.011	0.015	0.550	1	0.458	1.011	0.982	1.041
${\text{NO}}_{3}^{-}$	−0.096	0.120	0.642	1	0.423	0.908	0.718	1.149
${\text{PO}}_{4}^{3-}$	−36.198	15.829	5.230	1	0.022	0.000	0.000	0.006
${\text{SO}}_{4}^{2-}$	−0.043	0.053	0.663	1	0.416	1.044	0.941	1.159
Constant	−1.047	3.767	0.077	1	0.781	2.850		

## Discussion

### Enteric Viruses' Contamination

From the findings on virus detection rates across the six sampling points, the highest genome detection rates were for the points at the upper eastern side of the sampling stretch (A5 and A6). The two sampling points were situated in a region associated with possible sources of virus contamination via fecal pollution. Within the vicinity was a wastewater treatment plant as well as an informal settlement estate. The results were consistent with previous studies that have reported association of enteric viruses' contamination of surface waters and pollution from sewage such as by Zhu et al. (47). HAdV genome was not recovered from point A4, despite being the most stable and ubiquitous of the enteric virus (48). It is however noteworthy that this point is located in an area where small-scale agricultural activities are widely practiced, including livestock husbandry, but with limited human traffic compared to other parts of the sampling strip. Thus, because of the probability of minimal fecal exposure, HAdV genome may never have been detected. On the contrary, from this point, HEV was detected at a very low rate of 0.46%. The minimal detected could be attributed to transportation of the virus particles from distant reservoirs as a result of waves and constant water flow (18). Similarly, 0.93% of the HAdV positive samples were from point A3, but HEV was not detected from this middle stretch site. This difference in detection may be attributed to shift in the rate of human flow and related activities due to the existence of an open-air fish market within the vicinity. However, it is still possible to detect HEV at site A3 because pollution can be compounded by effluents being transported from far offsite potential sources of contamination through surface runoff (49).

Points A1 and A2 situated in the lower section of the sampling stretch each had a viral contamination rate of 0.46 % even though the surrounding area was characterized by intense urbanization and related activities such as water transportation, industrial, recreational and household water drawing. This decline in the occurrence of the viruses in this region could be associated with reduced fecal pollution compared to the other two sections. While these southern points were situated in an area associated with massive human traffic, their location may have decreased the chances of effluent contamination and potential viral contamination since they are about 3 km away from the key sources of fecal pollution such as the town's sewage treatment plant.

### Chemical Contaminants' Concentration

Apart from the main objective of the research which was to attempt to link the occurrence of HAdV and HEV from the water samples with concentration dynamics of the chemical contaminants, we also evaluated the chemical contaminants studied in comparison to the levels agreed by the WHO for safe water use. This has been informed by the fact that the exposure to higher proportions of certain chemicals such as the heavy metals in water supplies than the recommended limits may compromise the dependent population's health (50). Therefore, considering the fact that LV waters serve a population that is purely dependent on it for domestic, agricultural and drinking purposes (34), analysis of these contaminants was quite motivating.

Although the average concentration levels of all chemical pollutants were found to be minimal and below the WHO approved levels from all the sampling points that somehow qualifies the water as safe for human use, the findings showed a notable pattern of contamination levels. For example, the average concentrations of ${\text{PO}}_{4}^{3-}$ at point A4 were higher at 0.1811 mg/L than the rest of the sites even though this is still far below the normal WHO levels of 0.5 mg/L. The higher levels at point A4 may be due to the proximity of the site to some agricultural activities along the strip. The concentration of phosphates has been correlated with agricultural practices involving the use of agro-based fertilizers in other studies (51, 52). The higher concentration could also be attributed to runoffs from polluted industrial sections of the town to the lakefront. The mean Cd concentration was highest at points A6 (0.0006 mg/L) and A5 (0.0007 mg/L) respectively. The probable explanation for higher concentration from these sites may be related to the use of galvanized steel pipes for piping water to supply the settlement around site A6 from the old water treatment plant located about one km away (Figure 3). The galvanized steel water pipes are normally coated with some small amounts of Cd containing zinc (53). Corrosion of such pipes can therefore cause Cd and Zn to dissolve and contaminate the water sources. The higher concentrations from site A5 may also be linked to the sewage effluent and infiltration from the nearby sewerage treatment plant (53).

The average concentration of Fe was generally higher with a maximum value being above the standard WHO concentration of 0.3 mg/L at 0.8 mg/L. This could be attributed to pollution from the runoff from the contaminated sites. Previously, Homa Bay County had been home to an iron ore mining operation, at the slopes of Homa Hills overlooking the town and located at about 33 km away across the lake. According to CGOHB, (36) artisanal mining activities are still taking place at the site and therefore, leaching effects of residual deposits may still be possible. The average concentrations of Pb across all of the sampling sites were very low. The minimal concentrations may be due to washing off from the surrounding possible reservoir, such as the landfill near Asego Hill, about 3 km away. Environmental pollution of Pb has previously been associated with the presence of a landfill (54). The limited contaminations could also be due to leaching from water pipes (55). The minimum concentration for Cl^−^ was 5 mg/L, while the maximum was recorded from site A5 (20. 8611 mg/L). The slightly higher values recorded form sites A5 could be as a result of the impact of the pollution from the nearby sewage treatment plant located at the shoreline (56). Nitrate levels were within the WHO recommended limits, however, it should be noted that ${\text{NO}}_{3}^{-}$ concentrations are normally exacerbated by human and animal wastes as well as fertilizer usage (57). The study area is highly characterized by these potential sources of nitrate contamination and therefore high concentration values could still be reported in future.

### Relationship Between the Chemical Contaminats' and the Viruses' Contamination

It has been reported that the stability of viruses in an environment can be affected by different chemicals and, consequently, the concentration of such chemicals can be used to signal the probability of the occurrence of the viruses in the environment (14). From the present study, most of the chemical contaminants under consideration didn't correlate with virus detection. These findings are consistent with other similar studies conducted elsewhere such as by Vecchia et al. (15). The presence of viruses in water did not correlate with differences in various chemical contaminants (15). The apparent lack of significant correlation with most chemical contaminants may be attributed differences in rates of transformation of the chemicals in question (14). There were however some instances of interaction noted between some of the chemical contaminants and the viruses. For example, results from the Pearson correlation and logistic regression analyses between the heavy metals and the two sets of viruses indicated the existence of a positive relationship between concentration of HAdV and Cd, as well as between HEV, Pb and Fe.

These three heavy metals are some of the metals that have been reported to play an important role in maintaining the virus' structure and functions and may therefore affect the virus's stability and survival (17). They interact with the viruses by binding to the protein. They can alter important biochemical processes such as reverse transcription, translation regulation, RNA cleavage and catalytic activity. For example, in some viruses, Cd was reported to have catalytic activity while Iron was linked to oxidative processes. Pb has also been reported to be essential for polyprotein processing activation in certain viruses, such as hepatitis C (17, 58). Similarly, some studies have documented a lack of correlation on the relationship between the viruses and the examined anions in other parts of the world including South America and Europe. The anions; sulfate, nitrate, phosphate and fluoride did not correlate to the occurrence of the enteric viruses in surface waters (14, 31). Our study however showed a negative relationship between the concentration of HEV and phosphate. Phosphates have been reported to be involved in the interaction of proteins and nucleic acids (59) and thus can affect viral stability in the environment.

## Conclusions

The average concentrations of the 12 chemical contaminants considered in this study were found to be within the standards recommended by the WHO, thus qualifying the source waters to be safe with respect to those chemicals. However, the findings that the concentration of some of the chemicals such as Fe at concentrations above the limits allowed by WHO from some samples is a signal for possibility of substantial levels of contamination. Similarly, the general identification of heavy metals like Cd at some of the sampling points at slightly higher concentrations in general could be an indication for a possibility of potential undesirable level in the future due to continued accumulation. There were no significant negative associations between the variables except in the few highlighted cases. Therefore, there was no clear correlation to draw a fair inference to state categorically whether the chemicals studied reliably affect the stability of enteric viruses in the waters. Long-term multisampling approach and concentration of other potentially pathogenic viruses such as rotavirus are recommended in order to determine the true position of the use of chemical contaminants as possible indicators for the occurrence of enteric viruses in the environmental waters.

## Significance of the Study

In Sub-Saharan Africa data on different aspects of environmental monitoring are still minimal. Data from this study may be useful in informing the contact populations about the need to mitigate exposure to contaminants from environmental pollution control perspective. The data may inform remediation strategies to reduce chemical and viral exposure, and monitor pollution from multiple sources. Data on the association of the chemical contaminants with viruses' detection may contribute to the baseline for understanding the impact of interactions of viruses with different chemicals in the environment. The data could contribute to knowledge and understanding of strategies to reduce potentially pathogenic viral elements in the environment as well as for general bioremediation.

## Data Availability Statement

The datasets generated for this study can be found at the Kenyatta University repository http://ir-library.ku.ac.ke/handle/123456789/19905.

## Author Contributions

MW as the lead investigator formulated the topic and idea, collected information, wrote, and compiled the article. JM and OO were responsible for the supervision and review of the article from planning, dissemination, and execution. All authors contributed to the article and approved the submitted version.

## Conflict of Interest

The authors declare that the research was conducted in the absence of any commercial or financial relationships that could be construed as a potential conflict of interest.
